# Soil Carbon and Nitrogen Changes following Afforestation of Marginal Cropland across a Precipitation Gradient in Loess Plateau of China

**DOI:** 10.1371/journal.pone.0085426

**Published:** 2014-01-08

**Authors:** Ruiying Chang, Tiantian Jin, Yihe Lü, Guohua Liu, Bojie Fu

**Affiliations:** 1 Key Laboratory of Mountain Surface Processes and Ecological Regulation, Institute of Mountain Hazards and Environment, Chinese Academy of Sciences, Chengdu, Sichuan, China; 2 State Key Laboratory of Urban and Regional Ecology, Research Center for Eco-Environmental Sciences, Chinese Academy of Sciences, Beijing, China; 3 China Institute of Water Resources and Hydropower Research, Beijing, China; University of Maryland, United States of America

## Abstract

Cropland afforestation has been widely found to increase soil organic carbon (SOC) and soil total nitrogen (STN); however, the magnitudes of SOC and STN accumulation and regulating factors are less studied in dry, marginal lands, and therein the interaction between soil carbon and nitrogen is not well understood. We examined the changes in SOC and STN in younger (5–9-year-old) and older (25–30-year-old) black locust (*Robinia pseudoacacia* L., an N-fixing species) plantations that were established on former cropland along a precipitation gradient (380 to 650 mm) in the semi-arid Loess Plateau of China. The SOC and STN stocks of cropland and plantations increased linearly with precipitation increase, respectively, accompanying an increase in the plantation net primary productivity and the soil clay content along the increasing precipitation gradient. The SOC stock of cropland decreased in younger plantations and increased in older plantations after afforestation, and the amount of the initial loss of SOC during the younger plantations’ establishment increased with precipitation increasing. By contrast, the STN stock of cropland showed no decrease in the initial afforestation while tending to increase with plantation age, and the changes in STN were not related to precipitation. The changes in STN and SOC showed correlated and were precipitation-dependent following afforestation, displaying a higher relative gain of SOC to STN as precipitation decreased. Our results suggest that the afforestation of marginal cropland in Loess Plateau can have a significant effect on the accumulation of SOC and STN, and that precipitation has a significant effect on SOC accumulation but little effect on STN retention. The limitation effect of soil nitrogen on soil carbon accumulation is more limited in the drier area rather than in the wetter sites.

## Introduction

Clearing trees to create cropland can reduce soil organic carbon (SOC) significantly by up to 50% of the SOC in the initial forest [1], [2], [3], [4]. In contrast, the afforestation of previously arable land is generally found to sequester carbon (C) in the soil and play an important role in climate change mitigation [5], [6], [7], which was proposed as an effective method of C sequestration in Article 3.3 of the Kyoto Protocol in 1997. Subsequently, large-scale afforestation has been practiced worldwide in recent decades, especially in marginal land [8]. Thus, it is meaningful and necessary to study the process and mechanism of soil C changes and their causes during afforestation, especially when considering large-area plantation activities conducted in marginal land to mitigate CO_2_ emissions.

Soil C sequestration following afforestation has been the subject of a substantial body of research, which suggests that the direction and magnitude of SOC changes are determined by many factors and processes, such as climate, stand age and soil depth [6], [7], [9], [10], [11]. However, the effects of these factors on SOC sequestration are still controversial and unclear. Some reviews suggest that climate factors have a weak effect on soil C accumulation on a global scale [6], [11], whereas other meta-analyses indicate that climate plays an important role in influencing SOC during afforestation [9], [12]. Along a precipitation gradient (650 to 1450 mm), Berthrong *et al.* [10] found that SOC sequestration was significantly related to precipitation. In arid areas, precipitation is the most important ecological limiting factor and determines the net primary productivity (NPP); it may therefore have a significant effect on SOC, but such evidence is limited. The changes in soil C with stand age can vary, particularly in the initial stage of afforestation (<10 years). Soil C can decrease [7], [9], [10], [11], increase [10], [11], show no trend [13], [14], or produce a Covington curve [11] with stand age. It has been suggested that the different initial trend may depend on mean annual precipitation (MAP) [10], with soil C decreasing in wetter regions (MAP>1150 mm) but increasing in drier regions (MAP<1150 mm).

In contrast to SOC, the changes in soil nitrogen (N) following afforestation are poorly documented, even though soil N is strongly correlated with SOC, and SOC sequestration is controlled by the accumulation of soil N as a result of a stoichiometric relationship [15], [16]. In their meta-analysis, Li *et al.* [7] and Yang *et al.* [11] found a linear relationship between soil C and N following afforestation, and a 1 g N accretion was associated with a 7 to 13 g C accumulation. However, the interactions between soil C and N are less studied and less understood at the regional scale [17].

Plantations of N-fixing trees accumulate more soil C than non N-fixing forest [18], [19], [20]. An increase of 12 to 15 g C in the soils was found to be associated with an increase of a 1 g N in 19 case studies on N-fixing tree plantations [18]. The mechanisms behind the greater accumulation of soil C in N-fixing tree plantations may be the reduced decomposition of older soil C, increased formation of soil C, and a higher C input from vegetation, which is associated with the supply of N derived from fixation [18], [19], [20]. Yet the processes affecting soil C and N change once an N-fixing forest is established, and their relationship with stand age, remain unclear, especially in water-limited marginal lands. The N supply from decomposition is severely limited on dry land because of water limitations. As a result, increases in the external N supply from N-fixing tree plantations in arid areas may result in greater soil C than similar increases in moist regions.

Globally, most of the modern afforestation activities have occurred in China [8]. From 2000 to 2010, the area of afforestation and reforestation was approximately 4.9×10^6^ ha globally, and over 60% of the new forest area was in China [8]. Large areas of new forest have expanded during the modern period in China due to a series of ecological restoration programs, such as the Natural Forest Conservation Program and the Grain for Green project (GFG). The GFG is one of the most ambitious programs, initiated in 1999 in some areas and expanded in 2000 to the whole country. It aims to convert marginal, low-yield cropland slopes into woodland and grassland. The Loess Plateau, located in northwestern China, has a semi-arid continental climate (MAP ranges from approximately 650 mm in the southern Loess Plateau to approximately 250 mm in the north) and is the most important target for the GFGP due to its tremendous soil erosion. Between 2000 and 2008, the total area of the marginal cropland involved in the project was approximately 4.83×10^6^ ha, of which 3.84×10^5^ ha was converted into forest [21]. An N-fixing tree species, black locust (*Robinia pseudoacacia* L.), was one of the most commonly planted trees. Such large-scale plantation activity provides suitable conditions for studying the effects of afforestation on SOC and soil total nitrogen (STN) in marginal lands at a regional scale. The objectives of this study are to (1) detect the effects of MAP on soil C and N changes in younger (<10 years) and older (approximately 30 years) black locust stands in marginal cropland and (2) analyze the interaction between SOC accumulation and soil N accretion at the regional scale.

## Materials and Methods

### Site Description

All our field activities were conducted with the permission of the farmers who owned the land we tested, and we confirmed that the field studies did not involve any endangered or protected species.

Six sites (S1 to S6) were selected across the Loess Plateau (Fig. S1). The MAP increases from 380 mm in the northern site (S1) to 650 mm in the southern site (S6), with most precipitation occurring between June and August at all sites. The mean annual temperature is similar among the sites (Table 1). In all sites, the landform is typical loess and hilly, and the soil is a calcareous loamy soil (classified as an Entisol in USA soil taxonomy) and well drained [22]. The clay content of the soils increased significantly and linearly from 21.3% in the driest site (S1) to 38.2% in the wettest site (S6), whereas the soil sand content decreased significantly and linearly from 27.2% to 8.5% along this gradient (Fig. S2).

**Table 1:** Basic Descriptive Statistics of Six Plantation Sites Along a Precipitation Gradient.

Sites	Longitude/Latitude	Temperature(°C)	Precipitation(mm)	aCategory	Plantationage (years)	MeanDBH (cm)	Mean tree height (m)	Tree density(trees·ha^−1^)	bFine roots	cDescription
S1	110.22°/37.47°	9.5	380	Cropland (4)	0					
				Cropland (3)	0					
				Y (3)	7	6.5	5.2	900		
				A (3)	30	15.0	8.0	350		
				A (4)	30	14.0	11.0	700		
S2	108.11°/37.11°	8.5	420	Cropland (3)	0				Y (6)	
				Y (3)	8	5.0	3.5	1000		
				Y (4)	8	7.8	7.0	625	Y (7)	
				A (3)	30	15.0	7.0	800		
				A (5)	25	10.6	6.0	625	Y (9)	
S3	109.30°/36.92°	9.0	460	Cropland	0					[62]
				Cropland	0					[63]
				Cropland (4)	0				Y (8)	
				Y (5)	5	2.4	3.0	1200	Y (16)	
				Y (4)	8	6.1	6.5	1950	Y (16)	
				Y (3)	9	4.2	2.5	1100		
				A (3)	30	13.5	8.0	400		
				A (3)	30	11.0	8.0	1800		
				A (5)	30	16.1	10.0	500	Y (14)	
S4	109.53°/36.52°	8.7	530	Cropland	0					[64]
				Cropland	0					[23]
				Cropland	0					[65]
				Y (3)	5	6.3	7.0	2200		
				Y (3)	9	5.0	6.2	2700		
				Y (3)	9	5.1	5.8	2300		
				A (3)	25	9.0	6.8	1800		
				A (3)	30	10.5	6.5	2000		
				A (3)	30	18.0	7.0	1500		
S5	109.18°/36.07°	9.2	580	Cropland	0					[66]
				Cropland (5)	0				Y (10)	
				Y (3)	5	2.0	2.5	1400	Y (8)	
				Y (3)	6	5.0	7.0	5200		
				Y (3)	8	–	–	–		Tree census not detected
				Y (4)	8	5.6	7.0	2700	Y (16)	
				A (3)	26	12.0	12.0	1000		
				A (4)	28	13.2	15.0	1300		
				A (5)	30	7.1	7.5	1700	Y (17)	
S6	109.12°/35.33°	8.5	650	Cropland (4)	0					
				Cropland (5)	0				Y (10)	
				Y (3)	5	5.3	6.0	3000		
				Y (3)	6	5.2	6.5	3800		
				Y (6)	8	5.6	8.0	3200	Y (24)	
				Y (3)	9	5.8	8.0	4000		
				A (3)	25	9.2	12.0	2200		
				A (3)	30	14.3	13.0	1000		
				A (5)	30	13.8	13.0	1550	Y (20)	

^a^ Y and A indicate younger and older plantations, respectively. The number in the parentheses is the soil sample size in each stand.

^b^ Y (Yes) indicates that the fine root biomass was collected in this stand. The number in the parentheses is the sample size.

^c^ The data on soil organic carbon, total nitrogen content, and soil bulk density of some croplands were cited from other studies in the same site, and the data in the other cropland and forest stands were collected and analyzed in this paper. There was no tree census of the S5 forest stand, but the soil properties were measured.

In July and August of 2008, 2009, and 2010, a total of 17 younger (5 to 9 years at sample time) and 16 older (25 to 30 years at sample time) black locust plantation stands were chosen in the six sites, and in each site nearby, steep cropland stands also were chosen for comparison (Table 1). The aspect and angle of the cropland sites were similar to those of the corresponding black locust plantation stands. Most of the sloping croplands had been converted to other land use, especially since 1999 to 2000, so only a small amount of cropland existed, and only one or two cropland sites were selected in each location. Therefore, a few additional cropland data were cited and used from previous studies conducted at the same site (Table 1), which were believed to be reliable because the management history of cropland was similar and the soil C or N content of sloping cropland were found to be quite consistent in the same site in the Loess Plateau (e.g., Sun *et al.*, [23], Table 1).

We assumed there were no differences in the soil physicochemical properties between the black locust plantations and cropland sites before afforestation, and the changes in soil C and N afterward were primarily due to the plantation establishment. There are three lines of evidence to support these assumptions. First, all plantations were converted from long-term (at least 30 years) cultivated cropland, and soil C and N are generally thought to reach a constant value after long-term cultivation [3]. Second, the forest and cropland sites had similar histories and management before afforestation. Third, in each site, the soil characteristics, such as soil type (loess soil) and texture, were largely uniform [22], [24]. Furthermore, our assumption that there were no differences in soil C and N between pre-plantation and cropland was able to be strongly supported by the evidence that the soil C and N content of sloping cropland were consistent in the same site as shown above.

In each forest stand, black locust trees were planted in a regular pattern, and the same pre-planting site preparation, including disc trenching, was used across all the sites. After plantation, but prior to 2000, the older stands were occasionally thinned or used for livestock-grazing activities. Since 2000, there has been little human activity in the older plantations, and there has never been much in the younger stands. None of the stands has ever been fertilized. The annual fertilization application to cropland (farm manure or urea) was approximately 300 to 500 kg ha^−1^ across all sites. There has never been irrigation in the plantations or cropland.

### Field Measurements, Soil Sampling and Analysis

Generally, the area of the plantation stands was less than 0.5 ha, and only one plot (10 m×10 m or 20 m×10 m plot, depending on the area of the stand) was established in the center of each. The diameter at breast height (DBH), tree height, and tree density of the black locust trees in each plot were recorded (Table 1).

In each plot, locust trees were separated into three or four (dependent on the DBH values distribution) classes based on DBH, and one sample tree with the mean DBH value was selected per class. A 1 m×1 m subplot was established using the sample tree as a vertex, with three or four sample tree subplots in each plot. Sometimes, in addition to the sample tree subplots, another 1 m×1 m subplot (sometimes two) was randomly established in some plots. In total, three to six subplots were selected and were evenly distributed in each plot, and the sample size of each stand is shown in Table 1. In each subplot, an auger (3.3 cm diameter, 85 cm^3^) was used to collect soil samples for SOC and STN concentrations, and another soil auger (5.0 cm diameter, 100 cm^3^) was used to collect the soil samples for bulk density (BD). In all, there were three to six soil samples for SOC, STN, and BD taken from each plot, and all the samples were collected in increments of 0–10 cm and 10–20 cm. In some of the plots, soil cores (3.3 cm diameter, 85 cm^3^) were also collected to investigate the fine root (≤2 mm in diameter) biomass in each subplot, for a total of 6 to 24 soil cores in each plot (Table 1). Fine root samples were also partitioned into 0–10 cm and 10–20 cm increments. A detailed description of the fine root collection protocol is provided in Chang *et al*. [25].

In each cropland site, a 30 to 50 m transect was established at the center of the stand. Three to five 1 m×1 m subplots were established at approximately 10 m intervals along each transect. In each subplot, one soil core (3.3 cm diameter, 85 cm^3^) was collected to analyze SOC and STN concentrations. Another soil core was collected using a 100 cm^3^ cylindrical auger (5.0 cm diameter) for soil BD. In some of the cropland stands, two soil samples were collected in each subplot to assess the fine root biomass (Table 1). The soil samples for both physicochemical properties and fine root biomass were partitioned into 0–10 cm and 10–20 cm increments.

The SOC was measured using potassium-dichromate oxidation (titration of dichromate with ammonium ferrous sulfate), and the C content of the soil organic matter was assumed to be 0.58 [26]. The STN concentration was determined by the dry combustion method (1150°C) using a CN analyzer (Elementar Vario EL)(principle with thermal-conductivity detection for N_2_). Fine root samples were washed to collect the fine roots. Roots that were less than 2 mm in diameter were dried at 65°C to a constant mass in order to estimate the biomass. The soil samples for soil BD were dried at 105°C to a constant mass and weighed to determine the soil BD.

### Data and Statistical Analysis

#### Plantation aboveground biomass and fine root biomass

The aboveground biomass at the stand level in the plantations was calculated by multiplying the mean stem biomass by the tree density. The mean stem biomass was determined from the mean DBH based on the allometric relationship (between stem biomass and DBH) established in a previous study on black locust plantations [27]. The fine root biomass at stand level was calculated using the sample value.

#### SOC and STN stock estimation

The equivalent mass method (SOC stock calculated based on a reference soil mass) was not used in this study because there was a significant difference in soil BD in only a few plantation stands (four stands, 12.9% of total stands) and the corresponding cropland across the six sites. Thus the SOC or STN stock of each stand was calculated at a fixed depth as follows:
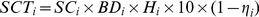
(1)where *SCT_i_* represents either the SOC or STN stock in layer 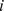 (in the top 10 cm layer or the 10–20 cm layer, Mg C·ha^−1^); 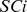 represents the SOC or STN concentration (g·kg^−1^) in layer 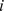; 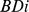 is the soil bulk density (g·cm^−3^) in layer 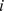; and 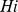 is the thickness (cm) of layer 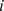. The term 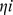 represents the volumetric percentage of particles with a size fraction >2 mm (rock fragments, %) in layer 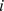, and this term was zero in each soil layer. The parameter 10 is a conversion factor.

#### SOC and STN stock changes

For each site, the mean SOC and STN of the cropland were calculated, and the absolute changes in SOC and STN stock (Mg C·ha^−1^) after planting were estimated as the difference between the SOC or STN stock in the tree plantation soil and the mean value of cropland soil, as in the following:
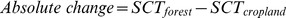
(2)where 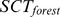 is the SOC or STN stock in the plantation (Mg C·ha^−1^), and 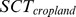 is the mean SOC or STN stock of the corresponding cropland for each site (Mg C·ha^−1^).

The relative SOC or STN stock change (%) after the establishment of tree plantations was calculated as follows:
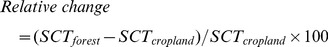
(3)where the mean 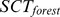 and 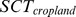 are shown above.

The C:N ratio change was used to represent the relative gain of SOC to STN following afforestation, and it was calculated as follows:
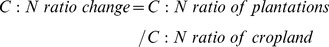
(4)where C:N ratio means SOC:STN.

### Statistical Analysis

A simple linear regression was used to test whether the tree density, tree height, and the plantation aboveground biomass increased with MAP and MAP had a significant effect on the SOC and STN stock and the C:N ratio (SOC to STN ratio) of the cropland and plantation stands and whether the MAP were significant predictors of the effects of afforestation on the SOC stock, STN stock and C:N ratio. In addition, the relationships between the SOC and STN stock changes during afforestation were tested using linear regression. The difference in the slope of the linear regression for different soil layers was tested (Z-test) using the following formula proposed in Paternoster *et al.* [28]:
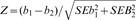
(5)where *b_1_* and *b*_2_ are the regression coefficient, and *SEb_1_* and *SEb_2_* are the coefficient variances of the two regressions, respectively.

A one-way ANOVA was used (Scheffé’s test was used if the data had equal variances and, if not, Tamhane’s T2 test was used) to compare the fine root biomass among the cropland and the younger and older plantations within each site, as well as among the sites for younger and older plantations. All analyses were performed in SPSS 11.0, based on a significance level of 0.05.

## Results

### Tree Census along the Precipitation Gradient

**Figure 1:**
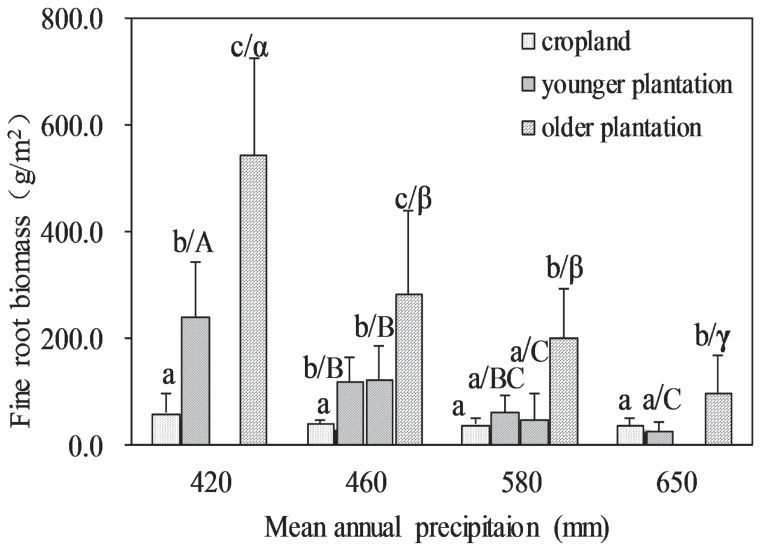
Comparison of fine root biomass among cropland and younger and older plantations in each site as well as among the younger and older plantations in four sites. Younger plantations are 5- to 9-year- old and older plantations are 25- to 30- year-old forests, respectively. The error bars represent the standard deviations of the means. The sample numbers are shown in Table 1. A different lowercase English letter is used for cropland and for younger and older plantations in the same site, and a different uppercase English letter represents younger plantations across the sites, whereas a different Greek letter is used for older plantations to indicate a significant difference at the 0.05 level.

Tree density increased significantly linearly with MAP increasing (R^2^ = 0.42, *p*<0.001), and tree height tended to increase along the gradient but with a low R^2^ (R^2^ = 0.12, *p* = 0.054). As the trend in tree density and height increased, the black locust plantation aboveground biomass also increased along the MAP gradient (R^2^ = 0.18, *p* = 0.015). In contrast, the fine root biomass decreased from the drier sites to wetter sites for younger and older plantations (Fig. 1).

With stand age, the fine root biomass of the younger plantations increased significantly in all four sites (Fig. 1), although the average age difference between younger and older plantations was approximately 22 years. In comparison to cropland, the fine root biomass of the younger plantations was significantly greater in the two drier sites (S2, MAP = 420 mm and S3, MAP = 460 mm), but not in the two wetter sites (S5, MAP = 580 mm and S6, MAP = 650 mm), and the fine root biomass of the older plantations was pronounced greater in all sites (Fig. 1).

### SOC, STN Stocks and C:N Ratio along the Precipitation Gradient

**Figure 2:**
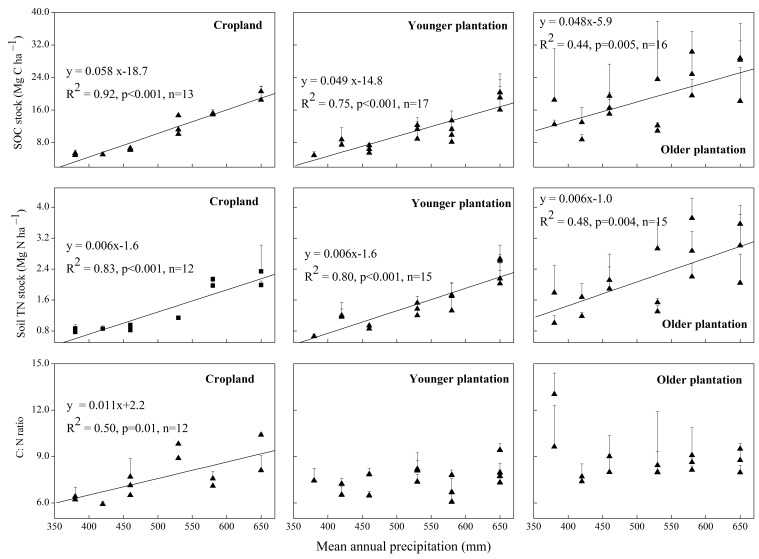
Relationship between mean annual precipitation and soil organic carbon (SOC), soil total nitrogen (STN) stock, and C:N ratio in cropland and younger and older plantations. Younger and older plantations are 5- to 9-year-old and 25- to 30-year-old forests, respectively. The error bars represent the standard deviations of the means (only shown in positive, n = three to six for each stand). The data for some cropland stands were cited from other studies (Table 1). Regressions were conducted for the top 20 cm of soil, and only significant regression models are displayed.

With an increase in MAP, the SOC stock of the top 20 cm layer of the cropland increased linearly from 5.2 Mg C·ha^–1^ in the driest site (S1) to 19.6 Mg C·ha^–1^ in the wettest site (S6; about a fourfold increase; R^2^ = 0.92, *p*<0.001), and the STN stock increased linearly from 0.8 Mg N·ha^–1^ at S1 to 2.2 Mg N·ha^–1^ at S6 (about a threefold increase; R^2^ = 0.83, *p*<0.001, Fig. 2). The greater sensitivity of SOC to MAP (compared with STN) led to a positive relationship between the C:N ratio of cropland and MAP (R^2^ = 0.50, *p* = 0.01, Fig. 2). For all plantations, the SOC and STN stocks in the top 20 cm layers also increased linearly with MAP (R^2^ = 0.75, *p*<0.001 for SOC of younger plantation; R^2^ = 0.80, *p*<0.001 for STN of younger plantation; R^2^ = 0.44, *p* = 0.005 for SOC of older plantation; and R^2^ = 0.48, *p* = 0.004 for STN of older plantation; Fig. 2). However, the regression slope was similar between SOC and STN for both younger and older plantations, resulting in a poor relationship between the C:N ratio of the plantations and MAP (Fig. 2).

### SOC and STN Stock Changes Following Afforestation along the Precipitation Gradient

**Figure 3:**
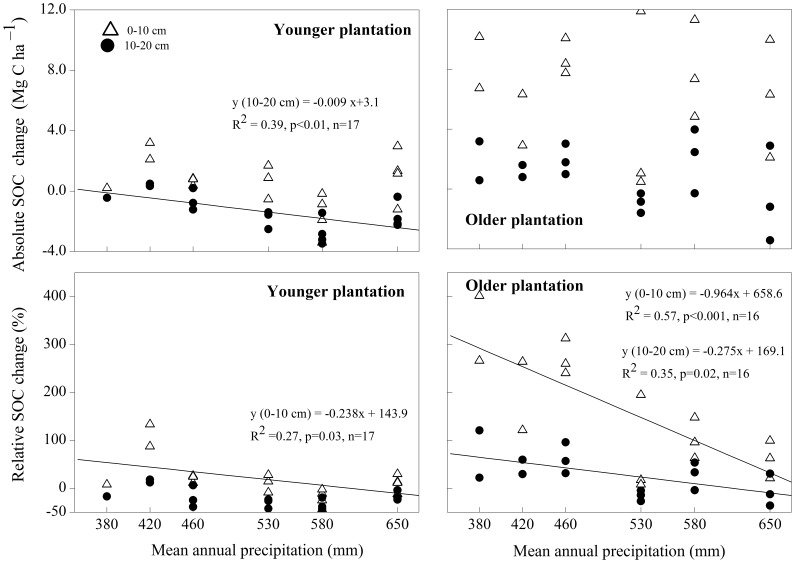
Relationship between mean annual precipitation and the absolute and relative change in soil organic carbon (SOC) in younger and older plantations. Younger and older plantations are 5- to 9-year-old and 25- to 30-year-old forests, respectively. Regressions were conducted separately for different soil layers, and only significant regression models are displayed. Positive values indicate an increase in the SOC stock due to afforestation, and negative values indicate a decrease.

The changes in SOC stock following the afforestation of cropland were dependent on soil depth and plantation age (Table 2, Fig. 3). For all the sites, the average absolute and relative SOC remained unchanged in the uppermost 10 cm and for the total 0–20 cm layer following afforestation, but decreased significantly (by 1.45 Mg C·ha^–1^ or 20.9%) in the lower 10–20 cm of soil (*p*<0.05, Table 2). In addition, the size of the decrease in absolute SOC in the 10–20 cm soil layer had a positive relationship with MAP (R^2^ = 0.39, *p*<0.01, Fig. 3), indicating that SOC stock decreased more in the wetter sites relative to the drier sites in the first plantation period.

**Table 2:** Changes in Soil Organic Carbon (SOC) and Soil Total Nitrogen (STN) Stocks in Younger (n = 17 and 15 for SOC and STN, Respectively) and Older Plantations (n = 16 and 15 for SOC and STN, Respectively) Established on Croplands.

Afforestationstages	Absolute SOC change(Mg C·ha^–1^)			Relative SOC change (%)			Absolute STN change(Mg N·ha^–1^)			Relative STN change (%)		
	0–10 cm	10–20 cm	0–20 cm	0–10 cm	10–20 cm	0–20 cm	0–10 cm	10–20 cm	0–20 cm	0–10 cm	10–20 cm	0–20 cm
Youngerplantation	0.43	−1.45**	−1.01	16.6	−20.9**	−2.3	0.12**	−0.08	0.04	21.5**	−9.8**	5.5
	(−0.44/1.31)	(−2.07/−0.82)	(−2.41/0.38)	(−4.7/37.8)	(−30.9/−10.9)	(−16.8/12.3)	(0.01/0.23)	(−0.17/0.01)	(−0.16/0.23)	(3.8/39.1)	(−19.5/−0.2)	(−7.1/18.2)
Olderplantation	6.70**	0.85	7.57**	161.1**	27.1**	93.8**	0.66**	0.11**	0.77**	109.4**	18.4**	63.5**
	(4.79/8.65)	(−0.23/1.93)	(4.85/10.29)	(97.5/224.4)	(3.9/50.4)	(52.3/135.4)	(0.41/0.91)	(0.01/0.21)	(0.45/1.10)	(63.8/154.9)	(5.0/31.8)	(35.8/91.2)

Significant at 0.05 level compared with the value of zero (one-sample T test).

The numbers in parentheses indicate the 95% confidence interval.

Younger and older plantations mean 5- to 9-year-old and 25- to 30-year-old plantations, respectively.

With an increase in plantation age, absolute and relative SOC increased significantly, by 6.70 Mg C ha^–1^ or 161.1% in the top 10 cm layer, and by 7.57 Mg C ha^–1^ or 93.8% in the total 0–20 cm layer (*p*<0.05 for both, Table 2). However, the absolute SOC showed little increase in the lower 10–20 cm soil layer (*p*>0.05, Table 2), suggesting that the initial decrease of SOC stock in this layer recovered to the level of cropland approximately 30 years after planting. For older plantations, the SOC stock in both the uppermost 10 cm layer and lower 10–20 cm increased more quickly in the drier sites than in the wetter sites, as shown by the negative linear relationship between MAP and the relative SOC changes (R^2^ = 0.57, *p*<0.01 for the top 10 cm, and R^2^ = 0.35, *p* = 0.02 for the 10–20 cm layer, Fig. 3). Moreover, the slope of the linear regression was greater for the top 10 cm than for the 10–20 cm layer (*p*<0.01, Fig. 3), implying that the relative SOC increased more rapidly in surface layers (0–10 cm) than in the subsurface layer (10–20 cm) as precipitation decreased.

**Figure 4:**
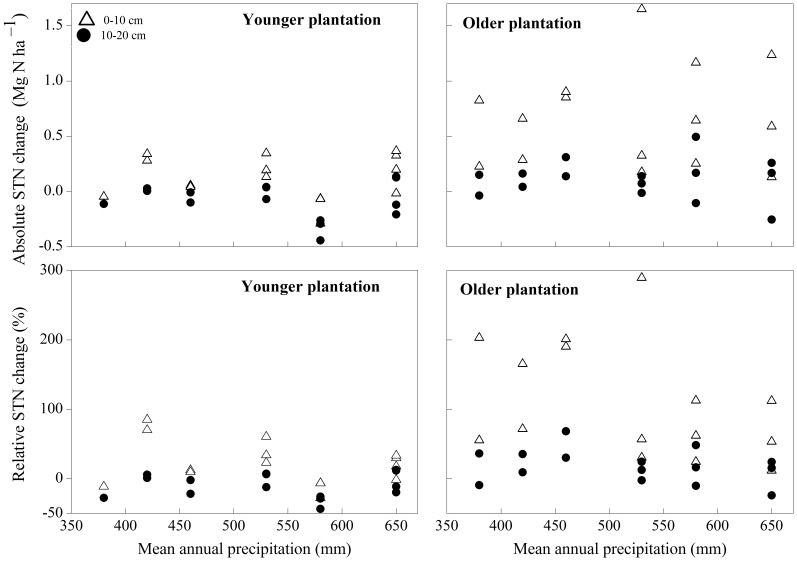
Association of absolute and relative change in soil total nitrogen (STN) in younger and older plantations with mean annual precipitation. Younger and older plantations mean 5- to 9-year-old and 25- to 30-year-old forests, respectively. Positive values indicate an increase in the STN stock due to afforestation, and negative values indicate a decrease. No significant association was found.

In contrast to SOC, neither absolute nor relative STN were related to MAP at any depth (Fig. 4). For all values of MAP, the absolute STN increased in the top 10 layer and remained constant in the lower 10–20 cm following afforestation, whereas there was no trend or a decrease in the absolute SOC in the upper and lower soil layers, respectively (Table 2). In older plantations, the absolute and relative STN had increased significantly in each soil layer, compared with the lack of change in absolute SOC in the lower 10–20 cm in younger plantations (Table 2). These results suggested that STN increased more quickly than SOC following cropland afforestation.

### Relationship between SOC and STN Changes Following Afforestation

**Figure 5:**
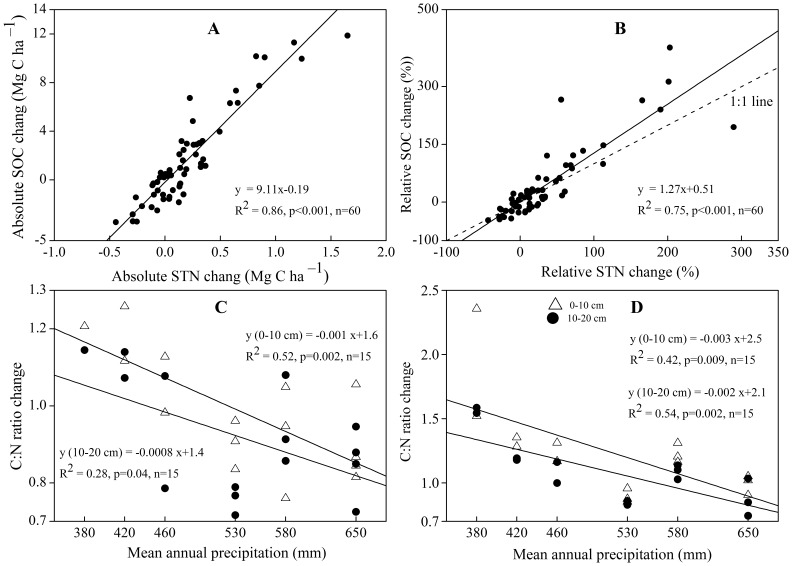
Relationship of changes in absolute soil organic carbon (SOC) and soil total nitrogen (STN) (A), changes in relative SOC and STN (B), and changes in the C:N ratio in younger (C) and older (D) plantations with mean annual precipitation. Younger and older plantations mean 5- to 9-year-old and 25- to 30-year-old forests, respectively. The dashed line in panel B indicates the 1∶1 line. Values greater than 1.0 in panels C and D indicate a relative gain of SOC to STN following afforestation, and lower values indicate the inverse.

Absolute and relative STN were found to increase significantly and linearly with increasing SOC (Figs. 5A, 5B), indicating that there is a close relationship between SOC and STN during afforestation at the regional scale. According to the relationship between absolute SOC and STN, an average gain of 1 g N was associated with a 9.1 g gain in C during afforestation (Fig. 5A). The slope (1.27) of the relationship between the relative SOC and STN was significantly greater than one (Fig. 5B), which implies that the relative STN exceeded SOC across the sites, confirming the relationship in average gains. However, the changes in SOC and STN during afforestation were dependent on precipitation (Figs. 5C, 5D). The negative linear relationship between the C:N ratio and MAP suggests that the relative gain of SOC to STN following afforestation was higher in drier lands and lower in wetter areas.

## Discussion

### SOC and STN Stocks along the Precipitation in the Loess Plateau

The SOC and STN stocks of younger and older plantations increased linearly as MAP increased from the north to the south of the Loess Plateau. These findings were consistent with the measured trend of SOC content in *Pinus tabulaeformis* plantations along the increasing precipitation gradient of the Loess Plateau [29] and the SOC stock’s tendency in the Nordic forest soils [30]. The positive relationship between precipitation and SOC and STN stocks could be related to an increase in NPP with precipitation [30], [31]. Also, the accompanying increase in tree density with precipitation is generally found to lead to a higher aboveground NPP and total belowground carbon allocation in forest [32]; however, the tree density has a nonsignificant effect on SOC stocks [32] because of the synchronous increase in soil CO_2_ efflux with tree density [33]. In addition, the greater SOC and STN stocks in the wetter Loess Plateau is associated with the higher clay content, which, it is suggested, plays a positive role in accumulating SOC and STN resulting from the chemical protection of SOC by bonds with clay surfaces and from physical protection through occlusion with aggregates [34], [35].

### SOC Changes Following Afforestation

Soil C is generally found to decrease in the first few years (>10 years) following afforestation, after which it increases with plantation age to pre-afforestation levels and then to net gains, which is indicated as a Covington curve [7], [9], [11]. Our findings showed a similar temporal pattern in absolute SOC, with an initial decrease in 8-year-old plantations followed by a return to the initial cropland SOC level in 30-year-old plantations in the 10–20 cm soil layer. The balance between soil C input and output determined the soil C pool. Accordingly, the initial decrease in SOC following afforestation indicates that the C input was too low to match the decomposition of SOC in younger plantations. A study using a natural^ 13^C abundance approach indicated that when cane (a C_4_ photosynthetic pathway) is planted with *Eucalyptus* (a C_3_ photosynthetic pathway), the new SOC_3_ (derived from the *Eucalyptus* planting) in the 10–20 cm layer increased by 1.8 Mg C/ha, whereas the older SOC_4_ (derived from cane) was lost at a rate of 3.2 Mg C/ha, resulting in a net C loss of 1.4 Mg C/ha in 10 to 13 years [14].

The initial accelerated SOC decomposition may be due in part to the intensive pre-planting site preparation, which included disc trenching in our study sites. In two reviews, Johnson [36] suggested that site preparation results in a net loss of soil C, although there is an accompanying increase in productivity, and Laganière *et al.* [6] suggested that low-intensity preparation (e.g., hand planting) may lead to a greater accumulation of SOC, and that high-intensity preparation (e.g., disc trenching) stops affecting the SOC level only 21 years after afforestation of cropland. Besides, the decomposition of older SOC is frequently found to be stimulated by the input of new liable C to soil in a short time scale, which is called a priming effect (PE) [37], [38], [39]. PE is suggested to be a global phenomenal, including in terrestrial and aquatic ecosystems [40], and the rhizosphere priming effect on SOM decomposition is from −70% to more than 380% [37], [39]. Thus the priming effect associated with the new C input, especially derived from fine roots during forest establishment, may in part induce the initial loss of old C from the arable soil.

The lose in absolute SOC in the 10–20 cm layer in younger stands increased with rising MAP, even though the NPP of vegetation is generally found to increase along the MAP gradient across the Loess Plateau [41]. This result provided evidence supporting the idea that the initial SOC loss during afforestation is more likely to occur in wetter soils other than in drier soils [10]. This condition may be ascribed to four causes. First, as predicted by the functional balance theory [42], [43], the fine root biomass of plantation stands was found to be greater in the drier sites where soil water availability was limited, unlike aboveground biomass. Fine roots have been suggested to play a more important role in soil C accumulation than the aboveground parts [44], [45]; therefore, the increased fine root biomass along the increasing MAP gradient may drive the trend in SOC change across the Loess Plateau. Second, the tree density increased with increasing MAP. Although tree density is suggested to have a limited effect on SOC changes during afforestation [6], higher tree density could entail an increase in the associated plantation disturbance. Thus the SOC loss resulting from the pre-planting preparations may be higher in the wetter sites. Third, microbial activity is suggested to be limited by the amount of soil water available [46], and soil respiration is generally found to be positively correlated with precipitation [47]. Thus the loss of soil C derived from decomposition would be less in the drier sites. Fourth, in the drier, infertile sites, the PE may also be constrained by limited soil water availability and decomposition substrate [38], [48], which also could reduce the SOC loss. Therefore, our results suggest that less SOC decomposition and higher belowground C input determined the initial SOC changes along the decreasing MAP gradient, rather than the NPP of afforestation.

After the initial decrease in SOC in younger plantations, soil C is usually found to increase with plantation age and to reach net gains in approximately 30 years [6], [7], [9], [10]. Based on global afforestation meta-data, Laganière *et al.* [6] found that the SOC stock decreased by 5.6% in young plantations (<10 years) but increased by 6.1% and 18.6% in medium-aged (10 to 30 years) and older (>30 years) plantations, respectively. Our findings showed a greater gain in SOC stock (161.1% and 93.8% in the top 10 cm and total 20 cm layers, respectively) in the 30-year-old plantations, which may be because the plantations were primarily N-fixing tree species.

In older plantations, there was a weak relationship between the change in absolute SOC stock and MAP. However, the negative relationship between the relative SOC changes and MAP suggests that the relative SOC gain was higher in drier sites than in the wetter sites. Berthrong et al. [10] also showed that more SOC accumulated in drier lands (MAP<1150 mm) than in wetter lands (MAP>1150 mm) following the afforestation of grassland. This current finding, in contrast to the trend of SOC during forest cultivation, indicates that SOC loss is greater in wetter regions than in drier regions [49], [50]. The greater relative increase in SOC in the drier sites was associated with a lower initial value of soil C in cropland. Thus, afforestation can increase SOC more rapidly and improve soil quality more in infertile arable lands than in fertile land, although it may have a similar effect on the accumulation capacity for SOC across the sites.

### STN Changes Following Afforestation

As with SOC, STN following afforestation also can show various temporal patterns [7], [10], [11], [13], [51]. In a global meta-analysis, STN stocks were generally found to follow a Covington curve, similar to the general trends in SOC, although the duration of the initial decrease in STN (50 years) is longer than that of SOC (30 years) [7]. However, our results showed no significant decrease in STN during early afforestation, whereas STN stocks tended to increase with plantation stand age. These findings were consistent with other studies of black locust plantations in the Loess Plateau [52], [53]. This increase in STN may, to a large extent, be due to the effect of biological nitrogen fixation by the black locust. Boring and Swank [54] reported that a 4-year-old black locust stand fixed 30 kg N/ha/yr during a one-year study period, and Rice *et al.* [55] found that there was 86 kg N/ha/yr of leaf litter returned to the soil by a 20- to 35-year-old black locust stand. These estimates exceed the average observed annual N accretion in our sites (4 to 9 kg N/ha/yr for younger stands and 26 to 31 kg N/ha/yr for older stands, respectively). Other N sources and mechanisms also may affect STN accumulation. For example, atmospheric N deposition (including dry and wet deposition) is often used to explain increasing STN during afforestation [7], [11]. However, few studies have examined the difference in atmospheric N deposition between plantation and cropland, and the N retained in the soil through deposition has rarely been quantified after afforestation. In addition, plantation areas may be less susceptible to soil erosion than croplands in the Loess Plateau [56]; therefore, the lower N loss from soil erosion may result in higher soil N after afforestation.

N mainly enters soil in three ways: atmospheric deposition, fertilizer, and N fixation. There was no N input from fertilizer in the plantations of the Loess Plateau. N inputs from atmospheric deposition are generally found to increase with MAP increase from the northern to southern areas of the Loess Plateau [57]. Meanwhile, biological N fixation is predicted to increase with tree density increase from the drier to wetter sites, and N-fixation is also suggested to be limited by drought conditions in the drier areas [58], [59]. Nevertheless, the changes in STN stock after afforestation were not found to be correlated with MAP in any age stand, which is in contrast to Berthrong *et al*.’s [10] finding that STN changes following afforestation were significantly related to MAP. This condition may result from higher N loss from plant uptake and mineralization and from less N gain from soil-erosion control in the wetter southern Loess Plateau. First, the aboveground biomass of plantation stands was positively correlated with MAP, and this higher aboveground biomass in the wetter sites may consume more soil N. Evidence shows that available inorganic soil N (NH_4_^+^ and NO_3_^−^) is lower with higher tree density (higher aboveground biomass) [32] and which may offset, in part, the benefit of greater N-fixation resulting from higher tree density. Second, stronger microbial activity and a more pronounced PE in the wetter environment (as discussed above) may lead to higher SOM mineralization and consequently higher N loss from gas (e.g., NH_3_, N_2_O). However, McCulley *et al*. [60] suggested the N cycling is tighter in the wetter grassland sites along a precipitation gradient in the Central Great Plains, and the net soil N mineralization did not increase along the precipitation gradient. Thus a more precise measurement of N mineralization in the forests across the Loess Plateau is needed to understand the changes in soil N. Third, soil erosion in cropland is more serious in the northern, drier sites relative to the southern, wetter areas [61], and the N gain derived from the reduced soil erosion in afforested sites may be more pronounced in the northern, more eroded sites.

### Soil C and N Interaction and the Implications

The loss of SOC is greater than that of STN when forests were converted into cropland, resulting in a lower C:N ratio in the cropland [1], [4]. In contrast, the accumulation rate of SOC is suggested to be higher than that of soil N after cropland afforestation [4], [7], [51]. In the present study, however, STN was found to increase more quickly than SOC following afforestation, as indicated by higher relative changes in STN. Nevertheless, the relative gains of SOC and STN also were precipitation dependent, with higher C gain (compared with N gain) in the drier lands and lower C gain in the wetter sites. Furthermore, SOC changes following afforestation were related to MAP, whereas STN changes were not. Therefore, C and N were more likely to be decoupled following marginal cropland afforestation in this semi-arid environment, and the changes in soil C and N were not synchronous at the regional scale, which is a proxy for precipitation. This finding implied that the mechanisms and factors controlling the accumulation rates were different for C and N, as suggested by McLauchlan [4].

As a result of the stoichiometric relationship between C and N, the terrestrial C (including soil C) sequestration is suggested to be determined by the availability of N and may be down-regulated as the supply of N is limited, as argued in the progressive N limitation theory [15]. In our study, however, the relative soil C gain to N retention increased when precipitation decreased, indicating a limited effect of N limitation on SOC accumulation in the drier area, at least in a relatively short plantation stage (during about 30 years). It seemed that an increase in soil N derived from N-fixing tree plantation in such N-limited arid lands could have a significant effect on soil C accumulation, and this effect was greater in the drier areas rather than in the wetter ones. Nevertheless, these results are based on a relatively short period of afforestation (30 years), and they may not hold up in the long term, although some studies suggest that forest ecosystems have an intrinsic ability to regulate the soil C and N accumulation and reduce N limitation in a relatively longer time [11]. So further investigations focused on the older stands in such arid areas are needed.

## Conclusion

Our study suggests that afforestation of marginal cropland could lead to a significant increase in SOC and STN stocks in a relatively short time (about 30 years) and underlines the effect of precipitation on SOC dynamics during afforestation in an arid environment. The results show that, along the increasing precipitation gradient, the initial absolute loss of SOC increased but the relative increase in SOC in the older ages decreased. This phenomenon is suggested to be associated with the greater soil C decomposition loss and the less belowground C input in the wetter sites, rather than the NPP of afforestation.

Unlike the SOC dynamics, there was no significant loss in STN during initial afforestation, and the changes in STN were not correlated with precipitation. This decoupled relationship between STN and SOC implies that a mechanism may have been regulating the effect of soil N retention on soil C accumulation to prevent progressive N limitation during forest development.

## Supporting Information

Figure S1 **Geographic locations of the study sites and croplands involved in Grain for Green project in China’s Loess Plateau.**
(TIF)

Figure S2 **Relationship between soil clay content of croplands and mean annual precipitation.** The error bars represent the standard deviations of the means (only shown in positive, n = three to five for each stand). The datum of one stand in S4 (with MAP of 530 mm) was cited from Li *et al.* [66].
(TIF)
