# Supplementary figures and images for: Soil Carbon and Nitrogen Changes following Afforestation of Marginal Cropland across a Precipitation Gradient in Loess Plateau of China
Source: PLoS One. 2014 Jan 8;9(1):e85426. doi: 10.1371/journal.pone.0085426 (PMC3885704; doi:10.1371/journal.pone.0085426)

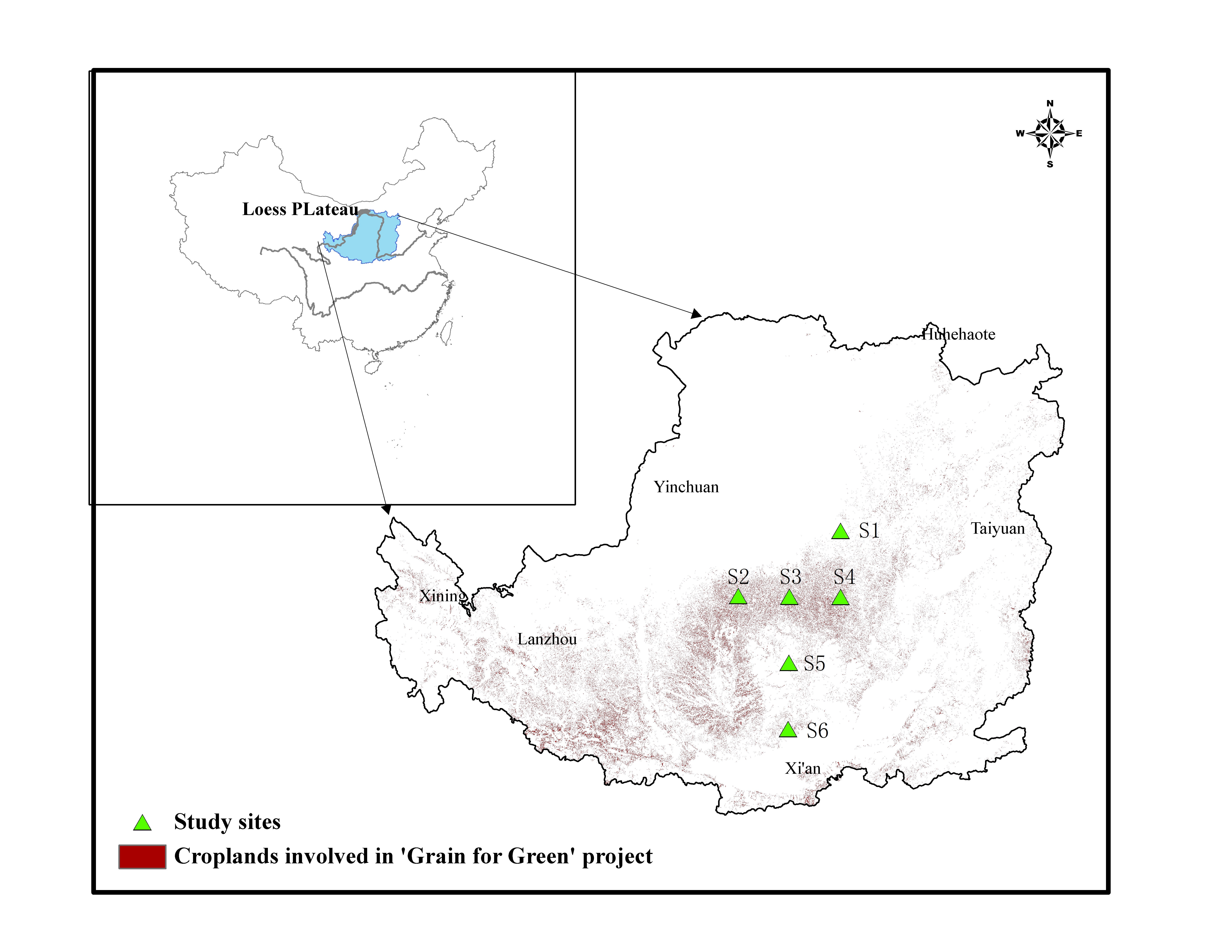

Supplement: Figure S1 — Geographic locations of the study sites and croplands involved in Grain for Green project in China’s Loess Plateau. (TIF) [file pone.0085426.s001.tif]
